# Editorial: Robots in assisted living environments: Enhancements, challenges and future perspectives

**DOI:** 10.3389/frobt.2023.1134462

**Published:** 2023-02-10

**Authors:** Alessandro Freddi, Andrea Monteriù, Vicente Ferreira De Lucena, Francesco Ferracuti, Sabrina Iarlori

**Affiliations:** ^1^ Department of Information Engineering, Università Politecnica delle Marche, Ancona, Italy; ^2^ Department of Electronics, Telecommunication, and Computer Engineering (FT), Federal University of Amazonas, Manaus, Amazonas, Brazil

**Keywords:** assistive robots, ambient assisted living (AAL), human in the loop (HITL), human robot interaction (HRI), healthcare robots

Medical and technological advances have enormously increased life expectancy in the last three decades. At the same time, in the previous year, the emergency caused by the COVID-19 pandemic obliged people to spend more time at home, often in social isolation and with limited access to routine healthcare. These evident transitions highlight the need to define a new paradigm in the future, where technology is used at home to improve the quality of life of people.

Ambient Assisted Living (AAL) technological solutions aim to improve people’s health, psycho-physical wellbeing, and independent living. Among such technologies, robots are now starting to play an important role and will probably play a fundamental role in the future.

This Research Topic aims to gather ideas, solutions and future perspectives on the use of robots in assisted living environments, both from the academic and professional world, to address the challenge of improving the quality of life of people at home. Beyond its survey aspects, this Research Topic also aims to collect relevant theoretical findings as well as challenging applications in various fields of robotics, with special attention to Human-Robot Interaction (HRI). Indeed, HRI is a key aspect for robots that operate close to or directly interact with people, such as in social robots, assistive robots or robot companions.

As a final goal, the Research Topic wants to provide an insight into the potential benefits of using robots in assisted living environments, and the current risks and difficulties that still limit the diffusion of robots as a consumer technology.

The Research Topic, which ended in November 2022, is a collection of six articles written by 38 international authors, and presents an overview of challenging AAL problems and innovative robotic solutions.

• Pinnelli investigates how the use of innovative technological devices to support the aging of frail elderly people does not necessarily correspond to an improvement in people’s quality of life. As such, the work focuses on the needs of strategic stakeholders and explores the factors that influence the adoption and diffusion of telemedicine devices by frail elderly people.
• Nieto Agraz et al. considers how technological advances in robotics have made its use possible in new application fields, like care, and may thus represent a viable solution in the future to the nursing labor shortage. Indeed, the study proposes a survey of robotic systems adopted for nursing care: a total of 133 robotic systems were identified and classified to provide a multidimensional view of the state of technology.
• Martini et al. presents the comparison of two web-based applications, namely RoboGen and RoboGen2, which healthcare professionals use as a medication management support system on healthcare/social robots for residents in a retirement village. The study shows that the proposed web-application has high usability scores and permits rapid medications with a good accuracy.
• Sorrentino et al. deals with the problem of developing assistive robots able to cope with a high variety of situations and contextualize their interactions according to the living contexts and habits (or preferences) of assisted people. The work proposes an original integration of AI-based features, and their deployment in a realistic assistive scenario, with the aim to define a human-in-the-loop continuous assistance procedure that helps clinicians in evaluating and managing patients, and to dynamically adapt robot behaviours to patients’ specific needs and interaction abilities.
• Ferracuti et al. proposes a human-in-the-loop approach, where the human can provide feedback to a specific robot, namely, a smart wheelchair, to augment its artificial sensory set, extending and improving its capabilities to detect and avoid obstacles. The architecture is preliminarily validated in simulation, using both a keyboard and a brain-computer interface to provide human feedback.
• Rulik et al. aims to design a multimodal control method for robotic self-assistance of individuals with disabilities in performing self-care tasks. A control framework for two interchangeable operating modes with a finger joystick and a chin joystick is developed and tested, where joysticks control a wheelchair and a wheelchair-mounted robotic arm.

The six articles of the Research Topic thus provide a synthetic but effective vision of robots in assisted living environments, ranging from specific assistive robotics applications, focusing on the key aspects of human-in-the-loop and adaptability, to an overview of robotic technologies, also considering the aspects of inclusiveness and user interfaces when developing assistive technologies.

